# Efficacy and safety evaluation of acupuncture therapy for patients with salpingitis in IVF-ET: A protocol of systematic review and meta-analysis

**DOI:** 10.1097/MD.0000000000024015

**Published:** 2021-01-15

**Authors:** Sixuan Li, Ying Ye, Zhaoxing Chen, Mao Zhao, Yuchang Jiang, Zhaodi Wang, Yong Jiang

**Affiliations:** Chengdu University of Traditional Chinese Medicine, School of Basic Medical Sciences.

**Keywords:** acupuncture, in vitro fertilization and embryo transfer, meta-analysis, protocol, salpingitis, systematic review

## Abstract

**Background:**

As an alternative for salpingitis in IVF-ET, acupuncture has gradually attracted the attention of clinicians based on the theory of syndrome differentiation and treatment of Chinese traditional medicine. However, due to the lack of evidence-based medical evidence, the author designed the program to evaluate the effectiveness and safety of acupuncture.

**Methods:**

From the beginning to August 2020, 7 electronic databases will be searched. Two of our researchers will independently conduct research selection, data extraction, and risk assessment of bias. We will use Review Manager 5.3 software for meta-analysis and heterogeneity assessment. In addition, we will use the grading of recommendations assessment, development, and evaluation to evaluate the evidence quality.

**Results:**

This study will demonstrate an evidence-based review of acupuncture for salpingitis in IVF-ET.

**Conclusion:**

The study will provide clear evidence to assess the effectiveness and side effects of acupuncture for salpingitis in IVF-ET.

**Trial registration number:**

INPLASY2020110125.

## 1 Introduction

Oviduct is one of the important parts of female reproductive system. The fertilized ovum is transported to the uterus through the oviduct. It can transport sperm and pick up ootid.^[1]^ Tubal infertility is one of the important causes of female infertility, accounting for 30% to 40%. Pelvic inflammatory infection is the most common cause of tubal lesions.

Tubal infertility is often caused by pelvic inflammation caused by ascending infection of pathogenic microorganisms.^[2–5]^ Salpingitis can lead to hydrosalpinx, adhesion, and even blockage, which is also the main reason for ectopic pregnancy. Salpingocyesis accounts for 95% of ectopic pregnancy.^[6]^ The more severe the salpingitis, the more severe the adhesion may be, and the more difficult it is to cure.^[7,8]^

The diagnosis of salpingitis often relies on hysterosalpingography (HSG), ultrasound (US) and laparoscopy, and there are also cases of auxiliary diagnosis of salpingitis combined with CT and MRI.^[9,10]^ In recent years, hysterosalpingo- contrast sonography (HyCoSy) has become a new technique for the diagnosis of tubal infertility due to its advantages of noninvasive, simple, convenient and low cost.^[11–13]^

Antibiotics, enzyme drugs and adrenocortical hormone drugs are often used to treat salpingitis. Among them, antibiotics can effectively eliminate pathogenic bacteria; Chymotrypsin and hyaluronidase can soften connective tissue and resist adhesion. Adrenocortical hormone drugs have good immunosuppressive and anti-inflammatory effects, which can block the local immune damage in the treatment and improve the pregnancy rate of patients. However, taking antibiotics for a long time is easy to lead to drug resistance, and the patient's condition is easy to relapse after drug withdrawal, which is not conducive to the recovery of prognosis. At this stage, clinical through uterine cavity drug injection, dredge fallopian tube and treat salpingitis. The combination of antibiotics, chymotrypsin, hyaluronidase and adrenocortical hormone can effectively improve tubal adhesion, dredge fallopian tube and increase pregnancy rate.^[14]^

Surgical treatment included hysteroscopy, radiomediated selective salpingography and recanalization. Hysteroscopy and laparoscopy have been widely used in the field of gynecology. Due to the anatomical characteristics of the female reproductive system, most of the causes of infertility can not be discovered by non-surgical examination. Some scholars pointed out that the total clinical effective rate of hysteroscopy and laparoscopy in the treatment of infertility was 96.67%, indicating that hysteroscopy and laparoscopy can clearly observe the specific conditions of the patients’ pelvic fallopian tubes, clarify the scope of lesions and give accurate diagnosis, and significantly improve the efficacy and pregnancy rate.^[15]^ Radiomediated selective salpingography and recanalization are mainly used for the treatment of proximal tubal obstruction. And it is used for the fallopian tube which can not be performed by liquid or contrast medium, so as to achieve the purpose of diagnosis and treatment. It was also reported that after selective salpingography and recanalization, the fallopian tube obstruction was improved, and the recanalization rate was 91.20%, which could effectively improve the degree of inflammation.^[16]^

However, the probability of spontaneous pregnancy after lysis of pelvic adhesion and fallopian tube recanalization depends on the location of tubal obstruction and the degree of fallopian tube lesions, and the pregnancy rate cannot be determined clearly. Some scholars believe that the ectopic pregnancy rate after laparoscopic surgery is significantly higher than the rate of IVF-ET, and IVF-ET also has a higher success rate,^[17]^ and can avoid the risk of ectopic pregnancy.

IVF-ET is a kind of technology that takes out oocyet and sperm from human body and fertilizes them in vitro. After developing into embryos, fertilized ovum can be transferred back to the uterus of the mother to achieve the purpose of pregnancy. Human in vitro fertilization and embryo transfer (IVF-ET) research is relatively late, but it develops rapidly. There are more and more people in need of assisted reproductive treatment worldwide. The reports show that more than 160 million^[18,19]^ IVF-ET provides an effective treatment method for successful pregnancy of infertility.^[20]^ With the rapid development of IVF-ET technology, the clinical pregnancy rate can reach 50%, but there is still a high spontaneous abortion rate of 18% to 35%.^[21]^

Although oviduct function is not dominant in IVF-ET, salpingitis may cause secondary inflammation of endometrium and pelvis, and will have a lasting and significant impact on IVF-ET. Therefore, in IVF-ET, the treatment of salpingitis is also an important step.

Some researchers thought that laparoscopic surgery for the diagnosis and treatment of pelvic lesions in patients with salpingitis has positive significance for IVF-ET.^[2]^ At present, most studies believe that hydrosalpinx caused by salpingitis will reduce the pregnancy rate of IVF-ET and increase the spontaneous abortion rate.^[22,23]^ Therefore, it is of great significance to make a clear diagnosis and treatment for clinical treatment of salpingitis.

Based on the basic theory of traditional Chinese medicine, acupuncture can balance the yin and Yang, promote local blood circulation and accelerate the absorption of inflammation. Acupuncture has been proved to be effective in improving the success rate of IVF-ET. However, the safety and effectiveness of acupuncture in the treatment of salpingitis in IVF-ET are still unclear. So we will organize, analyze, summarize studies that we could find on all databases about acupuncture for salpingitis in IVF-ET to provide a clear and significant evidence for clinicians.

## 2 Methods

### 2.1 Protocol register

This protocol had been registered on the INPLASY international prospective register of systematic reviews (INPLASY2020110125). We prepared the plan according to the preferred report project of systematic review and meta-analysis protocol guide.^[24]^ The final report will follow PRISMA recommendation to systematically review the report to incorporate the report into the network meta-analysis of healthcare interventions.^[25]^ the registration number is INPLASY2020110125, and the DOI number is 10.37766/inplasy2020.11.0125.

### 2.2 Ethics

Since the program does not require patient recruitment or collection of personal information, no further ethical approval is required.

### 2.3 Database search strategy

Use computer search and manual search for all published articles. We will search 7 databases, including 4 Chinese databases: VIP, Wanfang, CNKI, and the Chinese Biomedical Literature Database (CBM), and 3 English databases: the Web of Science, Pubmed, and Embase databases. All randomized controlled trials (RCT) of acupuncture used to treat salpingitis in IVF-ET will be searched until December 2020, regardless of reviews, protocols, animal experiments, case studies and clinical studies.

The specific search strategy will be formulated with a specific database (Table 1). Among them, the search strategy listed by authors had been supplemented by manually searching for relevant literature. At the same time, we also plan to manually search the published references of relevant systematic reviews. There is no date limit, country, publication status, or publication year limit.

**Table 1:** The search strategy in Pubmed.

1. Salpingitis [Mesh]
2. Salpingitis [Title/Abstract]
3. Inflammatory fallopian tube [Title/Abstract]
4. 1or2or3
5. IVF-ET [Title/Abstract]
6. In vitro fertilization embryo transfer [Title/Abstract]
7. Fertilization in vitro [Mesh]
8. In vitro fertilizations [Title/Abstract]
9. Test-tube fertilization [Title/Abstract]
10. Fertilization, Test-tube [Title/Abstract]
11. Fertilizations, Test-tube [Title/Abstract]
12. Test tube fertilization [Title/Abstract]
13. Test-tube fertilizations [Title/Abstract]
14. Fertilizations in vitro [Title/Abstract]
15. Test-tube babies [Title/Abstract]
16. Babies, Test-tube [Title/Abstract]
17. Baby, Test-tube [Title/Abstract]
18. Test tube babies [Title/Abstract]
19. Test-tube baby [Title/Abstract]
20. 5or6or7or8or9or10or11or12or13or14or15or16or17or18or19
21. Acupuncture Treatment [Mesh]
22. Acupuncture Treatments [Mesh]
23. Treatment, Acupuncture [Mesh]
24. Therapy, Acupuncture [Mesh]
25. 20or21or22or23or24
26. Randomized Controlled Trial [Publication Type]
27. Randomized [Title/Abstract]
28. 26or27
29. 4and20and25and28

### 2.4 Eligibility criteria and elimination criteria

#### 2.4.1 Type of studies

We will only include randomized controlled trials (RCTs) of acupuncture for salpingitis in IVF-ET, regardless of reviews, protocols, animal experiments, case studies, non-therapeutic clinical studies.

#### 2.4.2 Types of participants

All participants were clinically diagnosed with salpingitis in IVF-ET, without restrictions on the TCM classification of salpingitis in IVF-ET, such as age, sex, disease duration, and race. But some special patients will not be included even if they meet the clinical criteria for salpingitis in IVF-ET, such as pregnant or nursing women, people with severe heart, liver or lung disease, those with the history of major trauma surgery.

#### 2.4.3 Types of interventions

Observation group: Acupuncture is used alone or in combination with other treatment methods. The types of Acupuncture of TCM and methods of combination will be ignored.

Control group: Other treatments (including any other non- Chinese medicine treatment) or combined with fake acupuncture point.

#### 2.4.4 Type of outcomes

The outcome is based on the 2015 American Center for Disease Control and prevention on the diagnosis and treatment of pelvic inflammatory diseases and obstetrics and Obstetrics and Gynecology (8th Edition, People's Medical Publishing House of China), patients diagnosed with salpingitis may lead to hydrosalpinx, pus accumulation, adhesion, blockage and infertility, which should also be included in the category of salpingitis. The improvement of clinical symptoms (such as fever, abdominal tenderness or rebound pain, uterine and accessory tenderness, cervical lifting pain, etc) should be improved within 3 days after the treatment;^[26,27]^ the pregnancy rate after treatment should be more than 65%.^[28]^ The total effectiveness rate is a percentage, which is the ratio of the sum of the number of mitigators and effective people to the total number.

### 2.5 Studies selection and data collection

Literature management software (Endnote) for document management had been used. First, we will use the software to classify and organize documents, and remove duplicate documents based on the title and abstract. Second, the 2 researchers will independently screen relevant studies that meet the inclusion criteria based on the articles title, abstract, and keywords. Then, for uncertain research, we will download the full text for evaluation. The process will be completed independently by 2 researchers, and then the results will be cross-checked. And the 2 researchers will use pre-designed Microsoft Excel data extraction tables to independently extract the data in the study. The data items we plan to extract include:

1. Research characteristics (author, journal, year of publication, randomization method, blind method, etc).
2. Participants (sample size, age, disease duration, disease diagnosis criteria, etc).
3. Intervention (name of traditional Chinese medicine, type of treatment, dosage form, clinical dose, treatment process, etc).
4. Control (treatment type, treatment process, dosage form, clinical dose, etc).
5. Results (outcome, type of result measure, adverse event, etc).

**Figure 1:**
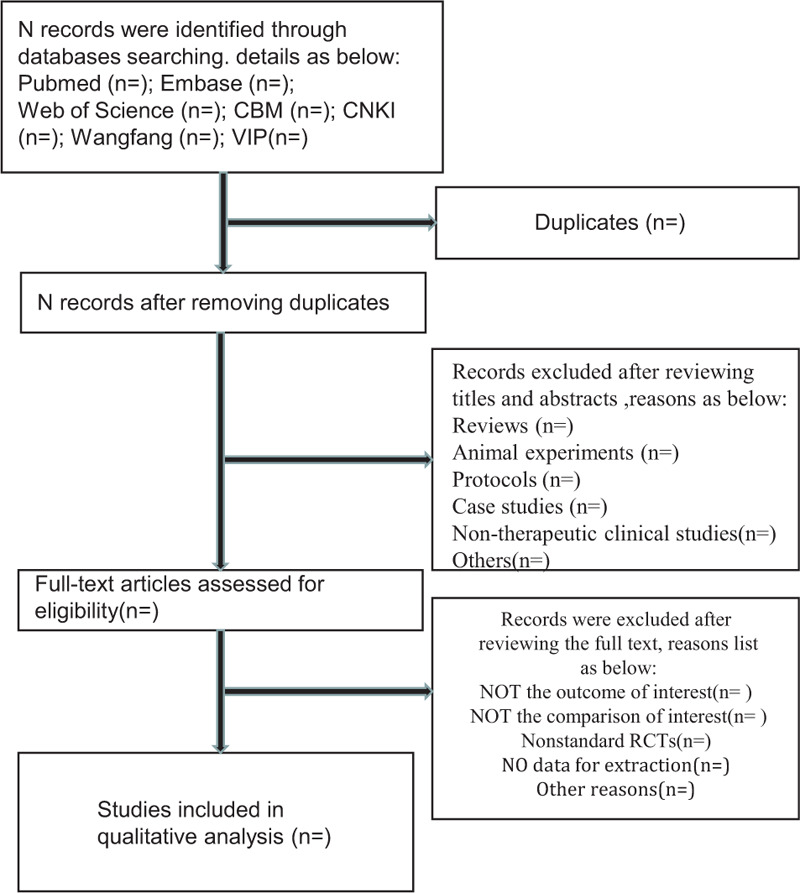
Flow diagram of the selection process.

At all steps, the 2 have to work independently and if there a disagreement between 2 people, it will be decided by a group. This selection process will follow the PRISMA guidelines as shown in Figure 1.

### 2.6 Dealing with missing data

We will get the missing data by contacting the study authors, and discuss the reasons, degree, nature, and how to deal with the missing data in each study. If data are still not available, we will only conduct a descriptive review of the study.

### 2.7 Assessment of risk of bias

Two researchers will evaluate the quality of RCTs by using the risk assessment tool recommended in Cochrane Handbook 5.3. This evaluation includes 6 factors: generation of random sequences, blinding of investigators and participants, blinding of study results, completeness of outcome data, selectivity in reporting of results, and other biases. If there are missing or unclear data, we will attempt to contact the original authors by email. If no reply is received or the authors have not saved the original data, we will analyze only the data that are useful in the literature or analyze the missing data in the discussion.

### 2.8 Literature quality assessment

The 2 researchers will evaluate the risk based on 4 areas: bias, inconsistency, indirectness, inaccuracy, and publication bias, and then grade the evaluate of results: high, moderate, low, and very low. We will use grading of recommendations assessment, development, and evaluation (GRADE) to assess the quality of evidence and the strength of the main result recommendation.^[29–31]^ Five factors can reduce the quality of evidence: study limitations (risk of bias), inconsistency, discontinuity, publication bias, and inaccuracy. And 3 factors can improve the quality of evidence: residual confusion, dose–response gradient, and large effects. The quality of evidence will be divided into 4 levels: extremely low, low, medium, and high. This step will be performed using GRADE software.

### 2.9 Data analysis

Revman 5.3 software will be used to combine and analyze the results of all the studies. This study involves bicategorical and continuous variables. The relative risk (RR) is used as an effect measure in the bicategorical variables and the mean differences (MD) in the continuous variables, and the software is able to obtain the point estimates and the 95% confidence interval (CI) for the 2. *I*^*2*^ is an important index for making the heterogeneity judgment. If *I*^*2*^ < 50%, a fixed effects model is used; if *I*^*2*^ ≧ 50%, a random effects model is used. For each combined analysis, the test of heterogeneity is measured using the cardinality statistic. If *I*^*2*^ ≧ 50%, substantial heterogeneity is considered to be present. If heterogeneity is present, we will analyze the cause through subgroup analysis and sensitivity analysis.

## 3 Discussion

In recent years, more and more reports and studies have confirmed that the influence of tubal inflammation on pelvic environment will reduce the success rate of IVF-ET. Acupuncture has been regarded a clinical effective treatment in improving the success rate of IVF-ET, but the treatment of salpingitis in IVF-ET has not been confirmed. The theory of acupuncture is from Chinese medicine, which can balance the bodies yin and yang by continuously stimulating acupuncture points. It can also stimulate the local pelvic blood circulation and inflammatory absorption, improve the pelvic blood supply and oxygen supply, and improve the success rate of IVF-ET. It is getting more and more attention as a TCM therapy. And acupuncture plays a role in osteoporosis, insomnia, obesity and magrine.^[32–34]^ Acupuncture has been a routine treatment in Chinese, but there is still no clear evidence of its effectiveness and safety onsalpingitis inIVF-ET. Therefore, it is significant to study these of acupuncture for salpingitis in IVF-ET. Improve the degree of salpingitis and increase the live rate of IVF-ET. We will refine this protocol to get useful results and provide Chinese solutions to medical professionals around the world.

## Author contributions

**Conceptualization:** Sixuan Li, Zhaoxing Chen, Zhaodi Wang.

**Data curation:** Sixuan Li.

**Funding acquisition:** Zhaodi Wang.

**Investigation:** Yong Jiang, Ying Ye.

**Methodology:** Mao Zhao, Yuchang Jiang.

**Project administration:** Yuchang Jiang, Zhaodi Wang.

**Software:** Zhaoxing Chen.

**Supervision:** Yong Jiang.

**Validation:** Ying Ye.

**Writing – original draft:** Sixuan Li, Yuchang Jiang.

**Writing – review & editing:** Yong Jiang, Sixuan Li.

## Abbreviations

Abbreviations: CI = confidence interval, CT = computed tomography, GRADE = grading of recommendations assessment, development, and evaluation, HSG = hysterosalpingography, IVF-ET = in vitro fertilization and embryo transfer, MRI = magnetic resonance imaging, NCCN = National Comprehensive Cancer Network, RCTs = randomized controlled trials, TCM = traditional Chinese medicine, US = ultrasound.

## References

[1] Jinqun H. Clinical value analysis of hysteroscopy combined with laparoscopy in the treatment of tubal infertility. J Qiqihar Univ Med 2016;37:2186–7.

[2] Ting L Jihui A Yu Z. The effect of laparoscopic surgery pretreatment on the outcome of IVF-ET. China Maternal Child Health 2015;24:4163–6.

[3] Qingzhi H. Efficacy observation of hysteroscopy combined with laparoscopy in the treatment of tubal infertility. Chin Med Innovation 2013;10:136–7.

[4] Xuemei B. Clinical value of hysteroscopy combined with laparoscopy in the treatment of tubal infertility. China Maternal Child Health Care 2012;27:5857–8.

[5] Chao Y. A brief introduction to the treatment of chronic salpingitis infertility with combination of Chinese and Western Medicine. J Practical Chin Med 2014;3:142–4.

[6] Lina Y. Study on the influence of different laparoscopic surgery on the second pregnancy. Jilin Med J 2015;36:1409–11.

[7] HaiYan H Yaqiong C Xiao C. Related factors and process of pelvic adhesion in infertile patients objective to investigate the effect of different degrees of salpingography on fallopian tube recanalization. Chin J Obstetrics and Gynecol 2018;47:69–71.

[8] Linghui H. Clinical efficacy analysis of Fuke Qianjin capsule combined with physiotherapy in the treatment of tubal adhesion. China Practical Med 2014;12:173–4.

[9] Fangyun B Fu L Yaping J. CT analysis of 31 cases of tubal inflammation. Chin J Integrated Chin Western Med Imaging 2017;15:94–6.

[10] Renrui W Qixia W Weiming H. MRI diagnosis of 29 cases of hydrosalpinx. Syst Med 2017;2:94–7.

[11] Cai C Qin Z Yuxi S. Application value of real-time three-dimensional hysterosalpingography in the diagnosis of hydrosalpinx. Chin J Integrated Chin Western Med Imaging 2019;17:600–2.

[12] Ahinko- Hakamaa K Huhtala H Tinkanen H. The validity of air and saline hysterosalpingo- contrast sonography in tubal patency investigation before insemination treatment. Eur J Obstet Gynecol 2007;132:83–7. 10.1016/j.ejogrb.2006.07.033 16952418

[13] Kupesic S Plavsic BM. 2D and 3D hysterosalpingo- contrast-sonography in the assessment of uterine cavity and tubal patencyJ. Eur J Obstet Gynecol 2007;133:64–9. 10.1016/j.ejogrb.2006.10.010 17329010

[14] Dan C Miaoshan L. Research progress of integrated traditional Chinese and Western medicine in the treatment of tubal inflammatory infertility. CJGMCM 2019;34:3057–8.

[15] Xiaoxia F. Clinical diagnosis and treatment effect of laparoscopy combined with hysteroscopy in female infertility patients. China Med Equipment 2017;32:177.

[16] Xiaohua H Li G Zhongmin W. Application of selective salpingography combined with recanalization in the treatment of tubal obstruction infertility. China Maternal Child Health Care 2016;31:5109–11.

[17] Gillett WR James C Jetta N. Removal of the ovarian surface epithelium from the rabbit ovary - a cause of adhesions following a standard injury. Hum Reprod 1994;9:497–500. 8006141 10.1093/oxfordjournals.humrep.a138534

[18] Dan P hanying Z Na L. Comparison of pregnancy outcomes between fresh transplantation and freeze thawed embryo transfer in patients with multi cycle IVF ET. J Reproductive Med 2010;29:1316–21.

[19] McLernon DJ Steyerberg EW Te Velde ER. Predicting the chances of a live birth after one or more complete cycles of in vitro fertilisation: population based study of linked cycle data from 113873 women. BMJ 2016;355:i5735. 27852632 10.1136/bmj.i5735 PMC5112178

[20] Xu F Wen L Ningxia S. Influencing factors of pregnancy failure in infertile patients undergoing assisted reproductive technology. Chin J Eugenics Genetics 2019;27:1131–2. 1135.

[21] Huiqin X Yanmei L Qingping K. Analysis and exploration of related factors of spontaneous abortion after assisted reproductive technology treatment. Shanxi Med J 2018;47:53–4.

[22] Chunmei Z Guipeng Liu Lingjuan Z. Laparoscopic treatment of hydrosalpinx for ovarian storage preparation function and effect of in vitro fertilization embryo transfer. J China Med Univ 2015;44:346–50. 356.

[23] Minggao C Lihua W Yan Z. Effect of embryo transfer outcome. J Int Radiol 2018;27:1173–6.

[24] Moher D Shamseer L Clarke M. Preferred reporting items for systematic review and meta-analysis protocols (PRISMA-P) 2015 statement. System Rev 2015;4:1. 25554246 10.1186/2046-4053-4-1 PMC4320440

[25] Hutton B Salanti G Caldwell DM. The PRISMA extension statement for reporting of systematic reviews incorporating network meta-analyses of health care interventions: checklist and explanations. Ann Intern Med 2015;162:777–84. 26030634 10.7326/M14-2385

[26] Xiaojuan L Aiping F Fengxia X. Interpretation of 2015 CDC guidelines for diagnosis and treatment of pelvic inflammatory diseases. J Int Obstet Gynecol 2015;42:674–5.

[27] M People's Medical Publishing House, Xing X Wenli G. Obstetrics and Gynecology. 2013;260-263.

[28] Ministry of health of the people's Re public of China. Specification of human assisted reproductive technology. Chin J Reproductive Health 2004;15:4–9.

[29] Andrews J Guyatt G Oxman AD. GRADE guidelines: 14. Going from evidence to recommendations: the significance and presentation of recommendations. J Clin Epidemiol 2013;66:719–25. 23312392 10.1016/j.jclinepi.2012.03.013

[30] Balshem H Helfanda M J Schunemann H. GRADE Guidelines: III Evidence quality classification. Chin J Evid Based Med 2011;11:451–5.

[31] Huang F Xie Y Zhao S. The effectiveness and safety of acupoint catgut embedding for the treatment of postmenopausal osteoporosis: a systematic review and meta-analysis. Evid Based Complement Alternat Med 2019;2673763. 31485243 10.1155/2019/2673763 PMC6710781

[32] Xu F Xuan LH Zhou HJ. Acupoint catgut embedding alleviates insomnia in different Chinese medicine syndrome types: a randomized controlled trial. Chin J Integr Med 2019;7:543–9. 10.1007/s11655-018-2770-3 30484016

[33] Chen IJ Yeh YH Hsu CH. Therapeutic effect of acupoint catgut embedding in abdominally obese women: a randomized, double-blind, placebo-controlled study. J Womens Health (Larchmt) 2018;6:782–90. 10.1089/jwh.2017.6542 29723106

[34] Xu J Zhang FQ Pei J. Acupuncture for migraine without aura: a systematic review and meta-analysis. J Integr Med 2018;16:312–21. 30007828 10.1016/j.joim.2018.06.002

